# Structural and effective connectivity in focal epilepsy

**DOI:** 10.1016/j.nicl.2017.12.020

**Published:** 2017-12-12

**Authors:** Christopher S. Parker, Jonathan D. Clayden, M. Jorge Cardoso, Roman Rodionov, John S. Duncan, Catherine Scott, Beate Diehl, Sebastien Ourselin

**Affiliations:** a Translational Imaging Group, Centre for Medical Image Computing, University College London, London, United Kingdom; b Developmental Imaging and Biophysics Unit, UCL Great Ormond Street Institute of Child Health, London, United Kingdom; c UCL Institute of Neurology, Department of Clinical and Experimental Epilepsy, Department of Clinical Neurophysiology, National Hospital for Neurology and Neurosurgery, London, United Kingdom

**Keywords:** Epilepsy, Seizure, Intracranial EEG, Cortico-cortical evoked potential, Diffusion MRI tractography, Graph theory

## Abstract

Patients with medically-refractory focal epilepsy may be candidates for neurosurgery and some may require placement of intracranial EEG electrodes to localise seizure onset. Assessing cerebral responses to single pulse electrical stimulation (SPES) may give diagnostically useful data. SPES produces cortico-cortical evoked potentials (CCEPs), which infer effective brain connectivity. Diffusion-weighted images and tractography may be used to estimate structural brain connectivity. This combination provides the opportunity to observe seizure onset and its propagation throughout the brain, spreading contiguously along the cortex explored with electrodes, or non-contiguously. We analysed CCEPs and diffusion tractography in seven focal epilepsy patients and reconstructed the effective and structural brain networks. We aimed to assess the inter-modal similarity of the networks at a large scale across the cortex, the effective and structural connectivity of the ictal-onset zone, and investigate potential mechanisms of non-contiguous seizure spread. We found a significant overlap between structural and effective networks. Effective network CCEP amplitude, baseline variation, and outward connectivity was higher at ictal-onset zones, while structural connection strength within the ictal-onset zone tended to be higher. These findings support the concept of hyperexcitable cortex being associated with seizure generation. The high prevalence of structural and effective connections from the ictal-onset zone to sites of non-contiguous spread suggests that macroscopic structural and effective connections are plausible routes for non-contiguous seizure spread.

## Highlights

• Inter-modal network agreement was higher than by chance and correlation was low.
• High CCEP amplitude, baseline variation and outdegree at the ictal-onset zone.
• Streamline density tended to be higher within the ictal-onset zone.
• High ictal-onset zone connectivity to early and late seizure spread sites.

## 1 Introduction

Focal epileptic seizures comprise localised areas of abnormal electrical activity which can subsequently spread to contiguous and non-contiguous brain areas. Approximately 30% of focal epilepsy patients are resistant to anti-epileptic drugs and in these cases resective surgery may be an option (Guerrini et al., 2003).

Pre-surgical evaluation aims to identify the area of seizure onset (the ictal-onset zone) and immediate spread to the brain regions which need to be resected to give the patient a good chance to become seizure free. In addition, identification of eloquent brain regions required for essential tasks, such as language, is important to understand the risks and benefits of surgery.

It may be challenging to localise the ictal-onset zone in focal epilepsy, particularly extra-temporal lobe epilepsies such as frontal lobe epilepsy (FLE). In FLE, there is incomplete understanding of how frontal lobe functional anatomy affects seizure semiology. Seizure freedom rates are 60–70% in temporal lobe epilepsies and less after extra-temporal resections (Téllez-Zenteno et al., 2005). Little is known about mechanisms of seizure propagation. Contiguous seizure spread, which is more commonly observed than non-contiguous seizure spread, is thought to occur through cortical layer V (Telfeian and Connors, 1998). However, intracranial EEG recordings may also demonstrate spread of seizure activity between non-contiguous areas (Duchowny et al., 2000, Turkdogan et al., 2005), ipsilateral or contralateral to the ictal-onset zone (Blume et al., 2001, Baumgartner et al., 1996, Lieb et al., 1987). It is unknown whether non-contiguous seizure spread may occur via direct cortico-cortical connection or indirectly via other cortical or sub-cortical sites.

Diffusion-weighted images and cortico-cortical evoked potentials (CCEPs) provide complementary information regarding connectivity of the presumed ictal-onset zone. Tractography, derived from diffusion imaging, estimates the paths of macroscopic white matter tracts, allowing reconstruction of the structural connectivity of cortical regions underlying intracranial electrodes. CCEPs, recorded on the intracranial EEG following single pulse electrical stimulation (SPES), indicate the presence of a functional tract from the stimulation to recording site, and therefore measure effective connectivity. Combining data from intracranial EEG, CCEPs and diffusion tractography may help characterise the connectivity of the ictal-onset zone and elucidate the mechanisms of non-contiguous seizure spread, improving understanding of epileptogenic networks and possibly leading to higher rates of seizure freedom following surgery.

Tractography studies in FLE have shown disturbances in structural connectivity of regions involved in epileptogenesis (Guye et al., 2003, Campos et al., 2015, Kovac et al., 2009). Ipsilateral and contralateral changes in microstructural indices of tracts closely related to suspected epilepsy pathology have been reported (Guye et al., 2003, Campos et al., 2015). Changes in functional but not structural global topology and a decoupling between global structural and functional connectivity has been found in childhood FLE (Vaessen et al., 2014).

Many studies have examined effective connectivity of epileptogenic networks using responses to SPES (Valentín et al., 2002, Valentín et al., 2005b, Valentín et al., 2005a, Flanagan et al., 2009, Iwasaki et al., 2010, Kokkinos et al., 2013, Boido et al., 2014). Delayed responses have been reported in seizure onset regions (Valentín et al., 2002, Valentín et al., 2005b, Flanagan et al., 2009). Higher amplitudes of the N1 CCEP component at ictal-onset electrode contacts compared to surrounding contacts (Iwasaki et al., 2010) was more pronounced in contacts showing repetitive spiking compared to paroxysmal fast patterns of seizure onset (Enatsu et al., 2012). Some SPES studies have demonstrated favourable surgical outcome in patients who had brain regions with abnormal responses to SPES resected (Valentín et al., 2005b, Flanagan et al., 2009).

Two studies have combined CCEPs and diffusion tractography (Conner et al., 2011, Swann et al., 2012), but did not examine connectivity of the ictal-onset zone or seizure spread.

In the current study, we reconstruct large-scale structural and effective connectivity networks by analysing all implanted electrodes, with three primary aims: (i) to assess the inter-modal similarity between structural and effective networks at a large scale across the cortex; (ii) to assess the potential for diffusion tractography and CCEPs to identify structural and effective connectivity markers of the ictal-onset zone and (iii) to examine the mechanisms of non-contiguous seizure spread using structural and effective networks.

## 2 Methods

### 2.1 Patients

Seven patients (4 male, mean age 34.6 years old, range 26–49 years old) were retrospectively selected from a large cohort of drug-resistant epilepsy patients who had undergone invasive intracranial monitoring at the National Hospital for Neurology and Neurosurgery (NHNN). These patients were the only individuals from the cohort who fitted the selection criteria. The criteria for patient selection were the availability of pre-implantation T1-weighted and diffusion-weighted MRI, post-implantation CT and T1-weighted MRI, and SPES. The study was approved by the local ethics committee and written informed consent was obtained. Five patients had frontal lobe epilepsy, two had parietal lobe epilepsy. Four had evidence of cortical dysplasia on MRI (patient details in Table S1).

### 2.2 Reconstructing structural and effective networks

Networks were reconstructed using the pipelines described in Supplementary Methods.

In brief, structural networks were reconstructed by recording the tractography streamline density between electrode ROIs. ROIs were propagated from CT to diffusion space via the post-implantation and pre-implantation structural MRI using linear and non-linear registration.

Effective connectivity networks were reconstructed by recording the absolute amplitude of significant CCEPs occurring between 12 ms and 250 ms post-stimulus, following SPES. This involved epoching, stimulation artefact reduction, visual exclusion of other artefacts, and identifying peaks in the CCEP. Inter-electrode effective connectivity was calculated as an average of the connectivity values from each stimulating electrode to the recording electrode. This conversion of data representations from the original format (stimulation electrode pair to recording electrode) to network format (stimulation electrode to recording electrode) allowed an analysis of CCEP connections with the native diffusion tractography network.

Networks were reconstructed for multiple connection features. Weighted effective networks were reconstructed for peak amplitude, latency and baseline standard deviation of the CCEP. Structural networks were reconstructed for streamline density. Both weighted and binary versions of the networks were analysed. Binary networks were produced by thresholding the weighted networks (see Supplementary Methods). As effective network weights were derived from the same set of significant CCEPs, the binary networks of different effective connectivity features are identical. For simplicity, the term effective networks will hereafter refer to the peak amplitude property of CCEPs, unless stated otherwise. Streamline density networks will be referred to as structural networks.

**Fig. 1:**
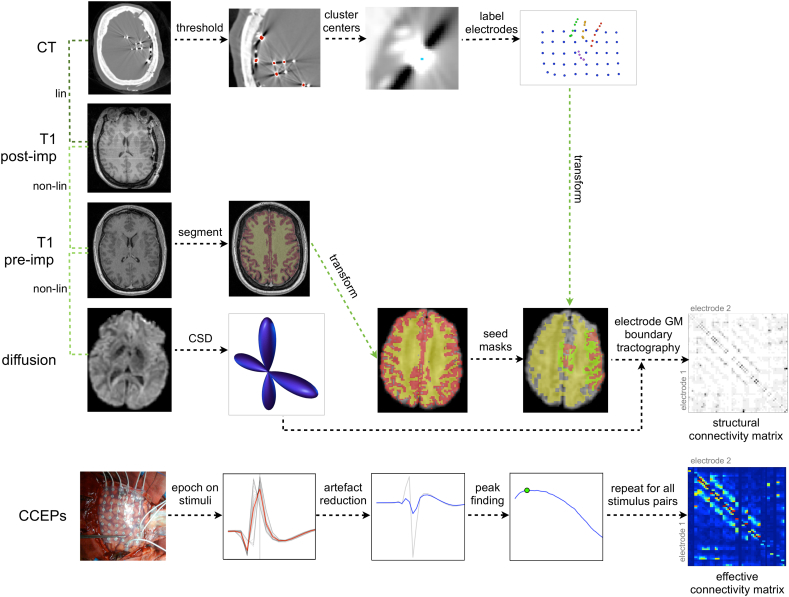
Overview of methods used to reconstruct structural and effective networks. Structural networks were reconstructed using probabilistic diffusion tractography between electrodes mapped from the CT to diffusion image via the pre- and post-implantation T1 images. Effective connectivity networks were reconstructed using peak amplitudes of the CCEP following SPES.

A summary of methods used to generate structural and effective networks is shown in Fig. 1. Reconstruction of structural connectivity networks required the pre-implantation diffusion-weighted and T1-weighted images in addition to the post-implantation T1-weighted and CT images. Reconstruction of effective connectivity networks required the original SPES data. Detailed methods for reconstructing structural and effective networks can be found in Supplementary Methods.

### 2.3 Graph theory analysis

Graph theoretical analysis was applied to binarised structural and effective networks, in order to compare the nodal connection topography between ictal-onset and non-ictal-onset contacts. Nodal graph theoretical measures calculated were indegree, outdegree, normalised indegree, normalised outdegree, clustering coefficient (Newman, 2003), centrality (Brandes, 2001) and reciprocity. Indegree and outdegree refer to the number of incoming and outgoing connections of a node, respectively. Normalised indegree and outdegree refer to the indegree and outdegree of each node normalised by the maximum given the number of nodes in the network. Reciprocity was calculated as the number of bidirectional connections as a fraction of the outdegree. Indegree, outdegree, clustering coefficient and centrality were calculated using the igraph package in R (Csardi and Nepusz, 2006). Reciprocity was calculated in R (R. C. Team, 2012).

### 2.4 Inter-modal agreement

Two comparison metrics were calculated:- the Jaccard Index, which measures the overlap in the binary structural and effective networks; and the Pearson correlation between structural and effective networks, which measures the correlation in the connection weights. The Jaccard Index was calculated on a patient-wise basis as the size of the set of intersecting connections divided by the size of the set of union connections.

### 2.5 Ictal-onset zone connectivity

During pre-surgical evaluation, each patient underwent video-EEG telemetry, recording multiple seizures. A set of typical seizures was identified for each patient by an experienced neurophysiologist (B.D).

Intracranial electrode contacts were classified by an experienced neurophysiologist (C·S) as being either located at the site of ictal-onset or not ictal. Ictal-onset contacts were those where seizure activity was first detected during the first typical seizure.

Seizure activity was defined as a clear ictal EEG pattern consisting of regular spikes, rhythmic sharp waves, spike-and-slow-wave complexes, sharp-and-slow-wave complexes, rhythmic delta or theta activities, sharpened delta or theta activities, or low-amplitude high-frequency activity in the beta range, as in (Alarcón et al., 1995).

**Fig. 2:**
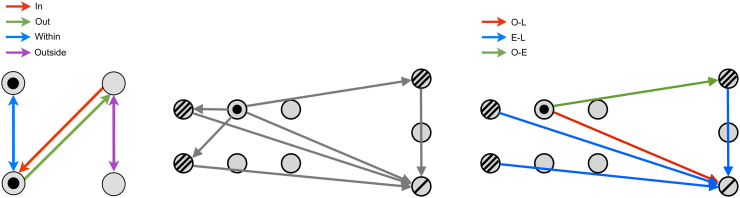
Illustrative examples of connection categories used in the ictal-onset and non-contiguous seizure spread analyses. Left: Ictal-onset connection categories. Ictal-onset contacts are filled circles. Red, green, blue and purple connections are those *in*, *out*, *within* or *outside* ictal-onset contacts, respectively. Center: A network containing contiguous and non-contiguous connections (grey lines) connecting ictal-onset contacts (filled circles), early propagative contacts (triple- lined circles) and late propagative contacts (single-lined circles). Right: Non-contiguous seizure spread connection categories. Contiguous connections were removed. Green, blue and red lines correspond to non-contiguous connections from onset to early, early to late, and onset to late contacts, respectively.

Network connections were classified as *in*- those entering an ictal-onset contact; *out*- those leaving an ictal-onset contact; *within*- those between ictal-onset contacts; and *outside*- those between non-ictal-onset contacts, as shown in Fig. 2. These categories permit observation of ictal-onset region connection subsets in context with those connections having no suspected involvement in epilepsy pathology. The features of interest (peak amplitude, latency, baseline standard deviation and streamline density) were then examined across the four connection categories on a pooled patient level for all CCEP connections. The nodal graph theoretical properties of ictal-onset and non-ictal-onset contacts were also compared on a pooled patient level.

### 2.6 Non-contiguous seizure spread

For each typical seizure, contacts were classified (C·S) as either ictal-onset- the contact where seizure activity is first detected; early propagative- contacts showing seizure activity within one second of seizure onset, and late propagative- contacts showing seizure activity between one and two seconds. Seizure activity was defined as described above.

Possible non-contiguous seizure spread connections were then identified from onset to early propagative contacts (O-E), onset to later propagative contacts (O-L), and early to later propagative contacts (E-L). Non- contiguous connections were those between contacts with distance > 11 mm, as determined using the electrode coordinates from the CT image. A distance of 11 mm was chosen as this considers the majority of row-wise adjacent grid and depth contacts as contiguous, while allowing some small displacement due to brain shift. Three non-contiguous seizure spread connection categories were therefore defined (Fig. 2). The prevalence of effective or structural connections in each of these three categories were calculated using the binary networks. Prevalence was compared to the density of the original binary and non-contiguous binary networks, as this represents the prevalence expected by chance. The distribution of peak amplitudes, peak latency and streamline density among non-contiguous seizure spread connection categories were obtained and compared to the distribution in the original networks. Analyses were performed on a pooled patient level.

## 3 Results

### 3.1 Structural and effective networks

Summaries of structural and effective networks are shown in Table 1 for all patients. The grand mean peak amplitude of effective networks across all patients was 163.8 ± 45.84 μV (mean ± s.d) whereas the grand mean streamline density of structural networks was 0.56 ± 0.064. The network density of binary networks was 0.169 ± 0.178 for effective networks and 0.109 ± 0.041 for structural networks. The mean global reciprocity of effective networks (0.34 ± 0.12) was < 1 as they are directed networks. The mean global reciprocity of structural networks was 1 as all connections are undirected.

**Table 1:** Summary of effective (Eff.) and structural (Str.) network properties across patients. Column abbreviations are as follows: Peakamp: mean peak amplitude. Lat: mean peak latency. Recip: global reciprocity. Streamdens: mean streamline density. JIrand: expected Jaccard Index between binary structural and effective networks due to random chance alone given the density of the networks. JI: observed Jaccard Index between binary structural and effective networks. Cor: Pearson correlation coefficient between weights of structural and effective networks. O: number of ictal-onset contacts. E: number of early seizure spread contacts. L: number of late seizure spread contacts.

Patient	Eff. Density	Eff. Peakamp (μV)	Eff. Lat (ms)	Eff. Recip	Str. Density	Str. Streamdens	JIrand	JI	Cor.	O	E	L
1	0.16	155.8 ± 71.6	77.70 ± 61.50	0.36	0.13	0.52 ± 0.51	0.08	0.21	0.18	1	4	1
2	0.04	202.00 ± 105.50	51.20 ± 42.20	0.22	0.09	0.62 ± 0.72	0.03	0.14	0.21	–	–	–
3	0.07	214.20 ± 131.50	48.60 ± 37.20	0.27	0.09	0.58 ± 0.58	0.04	0.17	0.05	2	4	12
4	0.15	134.20 ± 114.00	41.60 ± 29.20	0.44	0.06	0.50 ± 0.50	0.04	0.16	0.07	6	6	6
5	0.09	97.30 ± 85.20	42.30 ± 41.40	0.33	0.07	0.67 ± 0.89	0.04	0.20	0.06	2	10	4
6	0.11	211.00 ± 126.90	33.50 ± 22.20	0.22	0.15	0.50 ± 0.50	0.07	0.17	0.15	3	10	2
7	0.56	131.90 ± 104.30	31.80 ± 23.70	0.56	0.17	0.55 ± 0.59	0.15	0.18	0.17	2	4	2

### 3.2 Inter-modal agreement

**Fig. 3:**
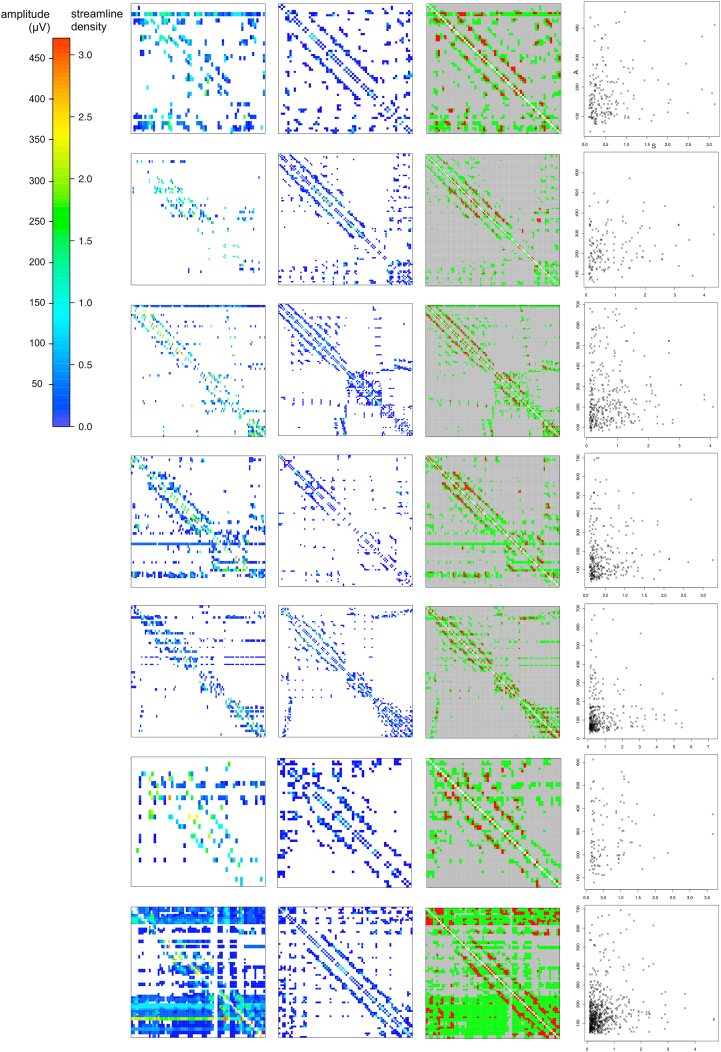
Effective and structural networks in all 7 patients. Left: Effective networks. Left-Center: Structural networks. Right-Center: Union (green) and intersection (red) of structural and effective networks. Right: Plot of effective network amplitude (A) and structural network streamline density (S). A high overlap but low correlation was observed between structural and effective networks.

**Fig. 4:**
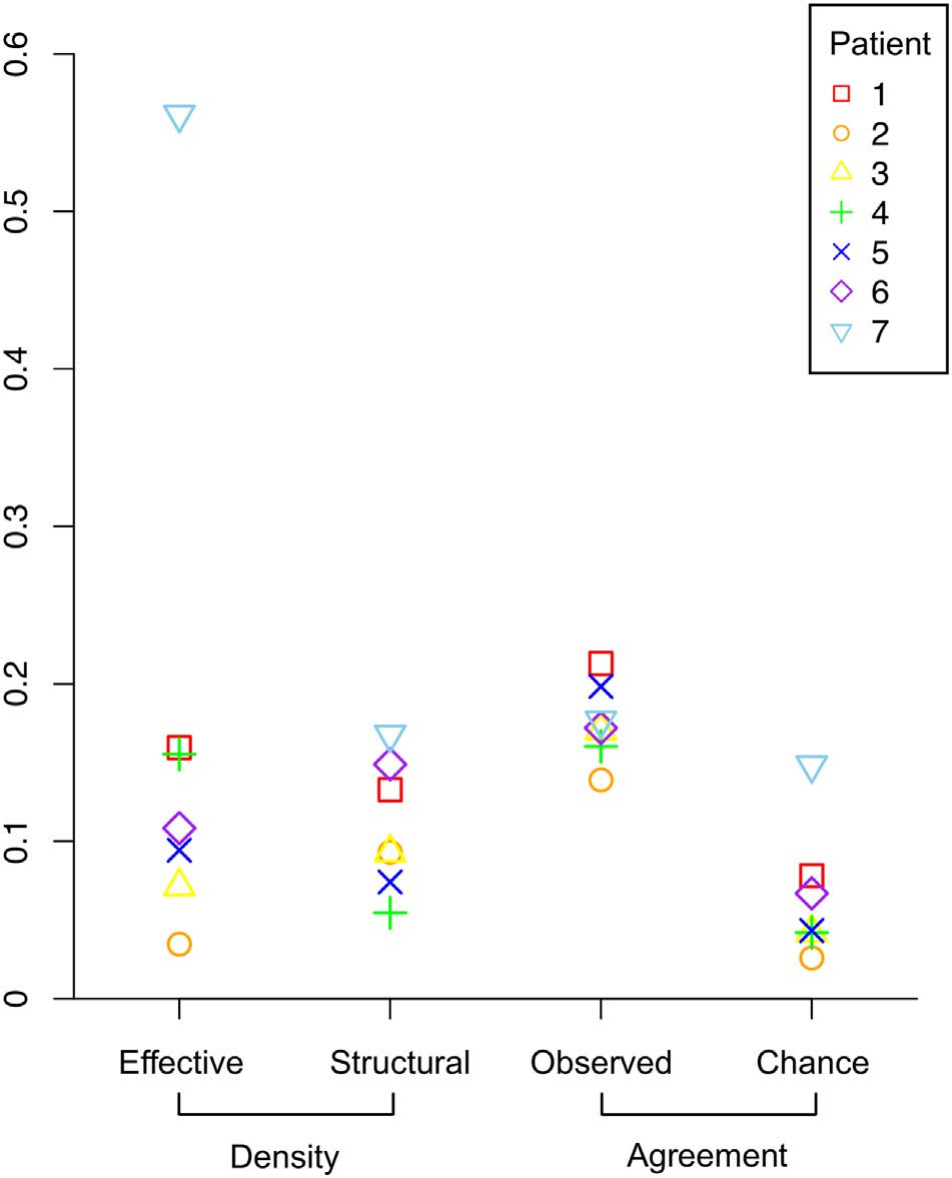
Agreement between binary effective and structural networks. The observed Jaccard index, measuring network similarity, was higher than expected given the density of the binary networks.

The mean overlap between the binary structural and effective networks, as measured by the Jaccard Index, was 0.176 ± 0.024. This was a higher overlap than expected by chance given the density of the networks (Fig. 3, Fig. 4). The Spearman correlation between the edge weights of structural and effective networks was low at ρ = 0.128 ± 0.066. (Table 1, Fig. 3).

### 3.3 Ictal-onset zone connectivity

The number of ictal-onset contacts for each patient is shown in Table 1. A total of 16 contacts were classified as ictal-onset (mean 2.7 ± 1.8 across patients). The patient with the lowest number of ictal-onset contacts was patient 1 who had one ictal-onset contact, while patient 4 had the most with six. Note that patient 2 had diffuse seizure onset patterns and was therefore not included in the ictal-onset connectivity analysis.

**Fig. 5:**
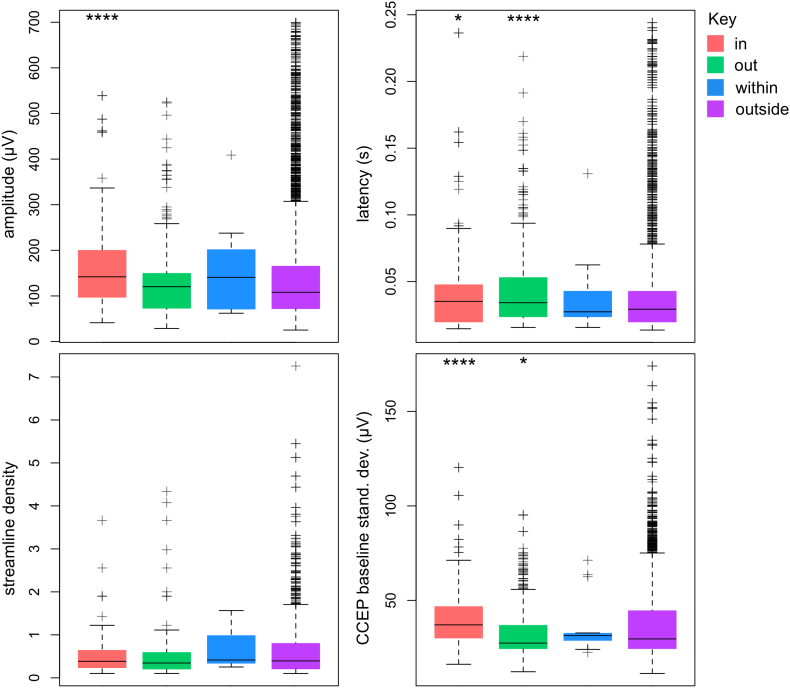
Effective connectivity of the ictal-onset zone (CCEP network amplitude, latency and baseline standard deviation and diffusion network streamline density) pooled across all patients. Shown are the median and interquartile range for four connection categories: *in* (connections towards ictal-onset contacts), *out* (connections away from ictal-onset contacts), *within* (connections between ictal-onset contacts) and *outside* (connections not involving ictal-onset contacts). Whiskers cover the range of data points no > 1.5 times the interquartile range. A higher CCEP amplitude and baseline standard deviation was observed for *in* connection categories, whereas a trend for higher distribution of CCEP amplitude and streamline density was found for *within* connections. For each connectivity measure a Wilcoxon rank sum test was performed to test for a difference in medians to the *outside* connection category. The significance of the test is indicated above each category (one, two, three and four stars indicate *p*-values less than or equal to 0.05, 0.01, 0.001 and 0.0001, respectively).

Ictal-onset connectivity category comparisons are presented for structural and effective connection features constrained along effective connections. Across all patients, a trend for higher peak amplitude at ictal-onset contacts was observed (Fig. 5). This was seen as a higher median peak amplitude for *in* (*p* ≤ 0.0001, Wilcoxon rank sum test) and *within* ictal-onset connection categories, compared to CCEPs recorded for *out* and *outside* connection categories. Higher baseline standard deviation of CCEPs was observed at ictal-onset contacts connecting from non-ictal-onset contacts, as shown by higher median baseline standard deviation for the *in* compared to *outside* connection category (*p* ≤ 0.0001). A trend of altered structural connectivity was found in the streamline density of effective connections between ictal-onset contacts, as shown by a higher median streamline density for the *within* connection category. The latency of CCEPs from the ictal-onset zone was higher compared to CCEPs between non-ictal-onset contacts (*p* ≤ 0.0001).

**Fig. 6:**
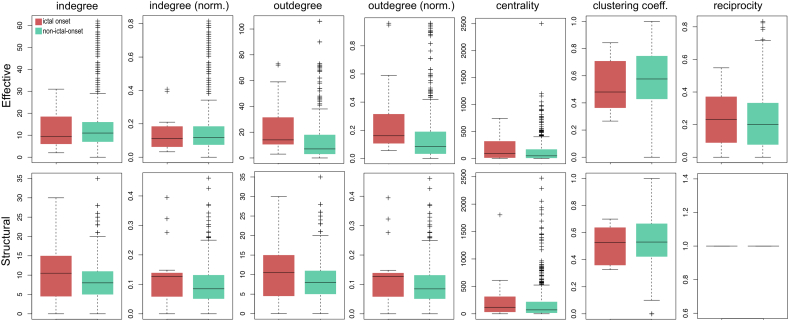
Nodal graph theoretical properties at ictal-onset compared to non-ictal-onset contacts. Node indegree, normalised indegree, outdegree, normalised outdegree, clustering coefficient, centrality and reciprocity are shown for structural and effective networks across all patients. Outdegree and normalised outdegree were higher at ictal-onset contacts in effective networks.

The majority of nodal graph theoretical properties showed little variability between ictal-onset and non-ictal-onset contacts. However, outdegree and normalised outdegree of ictal-onset contacts was higher in effective networks (Fig. 6).

### 3.4 Non-contiguous seizure spread

The number of contacts classified as early and late seizure spread sites varied across patients. The mean number of early seizure spread sites was 6.3 ± 2.9, whereas the mean number of late seizure spread sites was 4.5 ± 4.1 (Table 1). Patient 2 had diffuse seizure onset patterns and was therefore not included in the non-contiguous seizure spread analysis.

**Fig. 7:**
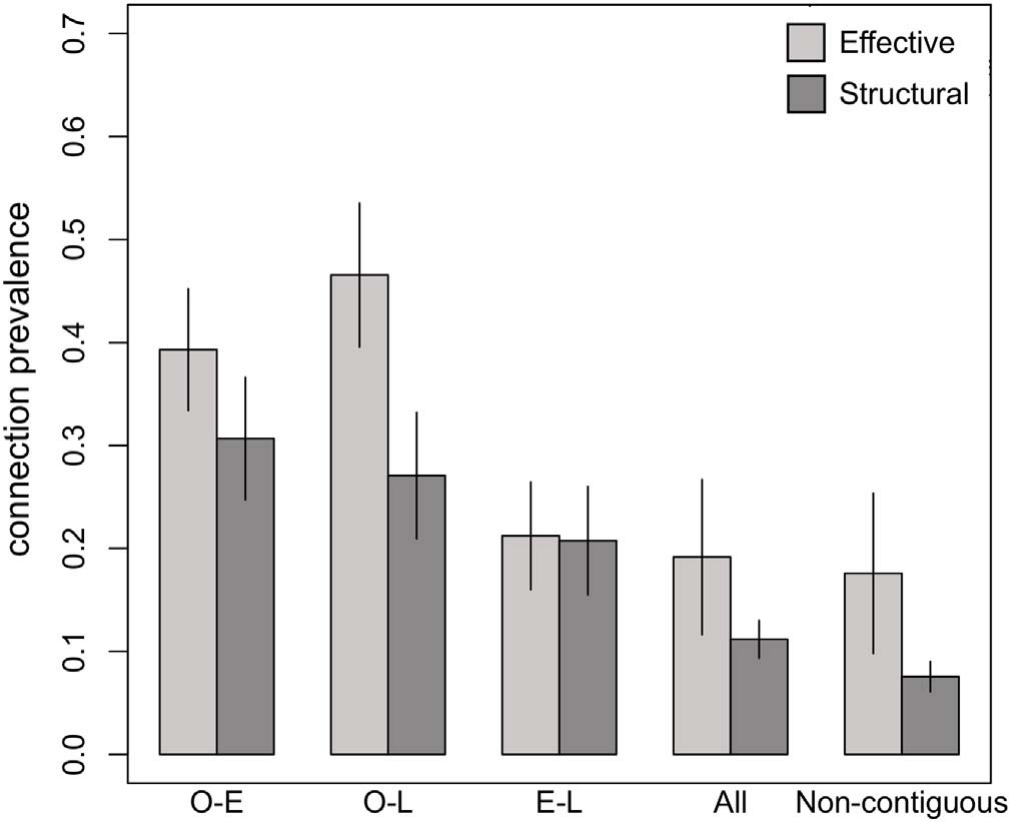
Prevalence (mean ± standard error across patients) of effective and structural binary network connections among all possible non-contiguous seizure spread connections (O-E: onset to early; O-L: onset to late; E-L: early to late). All structural non-contiguous seizure spread connection categories had a higher prevalence than expected compared to all network connections (All) and all non- contiguous connections. Effective networks had a higher prevalence of O-E and O-L connections, but not E-L connections.

The prevalence of both structural and effective connections among all possible non-contiguous seizure spread connections was higher than expected by chance (Non-contiguous connections, Fig. 7) for the O-E and O-L connection categories, whereas the prevalence of structural connections was also higher for the E-L category (Fig. 7). The chance prevalence was considered as the density of connections among all the possible non-contiguous connections, which was 0.176 ± 0.191 for effective networks and 0.076 ± 0.036 in structural networks.

Some trends in the weighted properties of non-contiguous seizure spread connections were observed in effective and structural networks. There was a trend for higher peak amplitude of O-E and E-L seizure spread connections in effective networks. The latency of O-E non-contiguous seizure spread connections was lower than the rest of the network and non-contiguous connections. Streamline density tended to be lower for O-L and E-L non-contiguous seizure spread connection categories.

Examples of non-contiguous seizure spread networks are shown in Fig. 9.

## 4 Discussion

We found a high degree of overlap between structural and effective networks, altered structural and effective connectivity of the ictal-onset zone, and evidence of structural and effective connections underlying non-contiguous seizure spread.

### 4.1 Inter-modal agreement

A high degree of overlap is expected between structural and effective networks as effective connections are constrained along structural connections; an axon is required to propagate a neural signal between brain regions. Some correlation in connection weights may also be expected as the density of connecting fibers (the structural network connection weight) may correspond to the number and synchrony of neurons activated at the remote site, which is directly proportional to CCEP peak amplitude (the effective connection weight).

Structural and effective networks had a high degree of overlap given the network densities, indicated by a high Jaccard Index, but the correlation in the connection weights was low. Two other studies have reported correspondence between CCEP and diffusion tractography connections (Conner et al., 2011, Swann et al., 2012).

Swann et al., (2012) found a high concordance in connectivity between pre-supplementary motor area (pre-SMA) and right inferior frontal gyrus (rIFG) using CCEPs, diffusion tractography and task-based functional MRI. Short latency CCEPs were elicited between the pre-SMA and rIFG sites which were also structurally connected (Swann et al., 2012). Conner et al., (2011) examined the correlation between the number of streamlines and CCEP amplitude in connections arising from Broca's area. They found a significant correlation R^2^ of 0.41. The higher correlation reported in Conner et al. could be because the connection weights of CCEPs and tracts arising from a single cortical area with a well characterised connection were examined- Broca's area is known to play a key role in language processing with connections to Wernicke's area via the arcuate fasciculus. In contrast, this study examined the entire set of cortical connections covered by the intracranial EEG, which included grid and depth contacts. This set of connections is likely to be more challenging to quantify through diffusion tractography or CCEPs than a single connection traversing a major white matter tract, and there may also be some false positive connections.

Loss of correlation between large-scale structural and functional networks has been previously reported in epilepsy. Zhang et al., (2011) found decreased structure-function correlation with epilepsy duration in idiopathic generalized epilepsy. Besseling et al., (2014) found lower structure-function correlation in rolandic epilepsy compared to controls, and Vaessen et al., (2014) found an absence of increase in correlation between structural and functional networks with age in childhood frontal lobe epilepsy, which was present in controls. These studies suggest that although structure-function correlation is typically present in epilepsy patients, it is lower in some forms of epilepsy.

The low correlation between structural and effective networks observed in this study may be due to a combined effect of disease-related loss of structure-function correlation, and imperfections in reconstructing structural and effective networks at a large scale across the cortex. Possible sources of error in structural network reconstruction include imperfect electrode localisation and relative inability to track short-range connections with high curvature, whereas possible sources of error in effective network reconstruction include remaining contamination of stimulation artefact. Other measures of connection weight are available for both structural and effective networks which may give higher correlation. For example, structural networks may be weighted by tract volume (Vaessen et al., 2011), fractional anisotropy along the tract (Lin et al., 2008), or combinations of these measures which can also penalise shorter distance connections (Li et al., 1894). Effective networks may be weighted by the negative amplitude of the evoked potential (Araki et al., 2015) or the root-mean-squared ratio of the response to the baseline (Lega et al., 2015).

Some studies have shown that lower stimulation amplitude activates a smaller volume of neural tissue (Ranck Jr, 1975) and elicits lower amplitudes of evoked response (Entz et al., 2014). In our study stimulations below 4 mA were applied between electrodes with closer spacing in order to maintain a similar charge density and neuronal activation to typically spaced electrodes (McCreery et al., 1990, Nathan et al., 1993). Lower amplitude stimulations were also applied following after-discharges or when clinical signs and symptoms were observed during SPES. Stimulation amplitude was not included in the effective connectivity weight calculation, which may have resulted in reduced amplitude of a small number of evoked responses. Further research to quantify the relationship between stimulation amplitude and evoked response will allow a more accurate definition of effective connectivity weight and facilitate greater comparability of CCEP data acquired with different stimulation amplitudes.

### 4.2 Ictal-onset zone connectivity

Previous studies have observed alterations in structural and effective connectivity of the ictal-onset zone (Guye et al., 2003, Campos et al., 2015, Valentín et al., 2002, Valentín et al., 2005b) in addition to regions outside of the ictal-onset zone (Lin et al., 2008, Zhang et al., 2011, Xu et al., 2014) in focal epilepsy. In this study, a higher amplitude of CCEPs was found at the ictal-onset zone. Furthermore, a higher baseline standard deviation was observed at the ictal-onset zone in the intracranial EEG of epochs containing significant CCEP peaks when stimulating outside of the ictal-onset zone. Previous CCEP studies have demonstrated a higher prevalence of delayed responses (Valentín et al., 2002, Valentín et al., 2005b, Flanagan et al., 2009) in addition to a higher amplitude of CCEPs at ictal-onset zones (Iwasaki et al., 2010, Enatsu et al., 2012). The CCEP findings are consistent with hyperexcitable cortex underlying the ictal-onset zone and promoting epileptic activity. Hyperexcitability in epilepsy may be due to a number of factors such as the presence of pathological tissue (e.g. lesions, cortical dysplasia), down-regulation of local inhibitory circuits or up-regulation of excitatory circuits (Goldberg and Coulter, 2013).

Cortical dysplasia was highly prevalent in the study cohort. Cortical dysplasia is a congenital abnormality of neuronal migration, resulting in abnormal grey matter. Four of seven patients (patient 1, 2, 4 and 6) had type IIb focal cortical dysplasia (Blümcke et al., 2011). In these patients the cortical dysplastic tissue was located in approximately the same brain region as the contacts recording ictal onset, although exact anatomical localisation of cortical dysplastic tissue was not performed. Focal type IIb cortical dysplasia is associated with cytomegalic dysmorphic neurons and balloon cells, distortion of the laminar structure of the cortical grey matter, and reduction in myelin of the underlying white matter (Blümcke et al., 2011). Such pathology gives rise to hyperexcitable abnormal neural tissue (Turkdogan et al., 2005). More rapid propagation of seizure activity has been found in patients with dysplastic lesions than those with non-dysplastic lesions. Also, the majority of patients with focal cortical dysplasia had fast frequencies at seizure onset compared to patients who did not have cortical dysplasia. The latter had more repetitive spiking at seizure onset (Turkdogan et al., 2005). There is a possibility that cortical dysplastic tissue resulted in higher amplitude of CCEPs. Further work is needed to differentiate between the contribution of the pathological finding of cortical dysplasia and the hyperexcitability of the cortex per se.

### 4.3 Non-contiguous seizure spread

Contiguous seizure spread is more commonly observed than non-contiguous seizure spread. Contiguous spread is thought to occur through cortical layer V (Telfeian and Connors, 1998), but the mechanisms of non-contiguous seizure spread are unclear. This study found a higher prevalence of non-contiguous effective connections from onset to early and onset to late seizure spread sites than expected by chance, given the density of the non-contiguous connections. In addition, structural connections from onset to early, onset to late, and early to later propagative sites were all more highly prevalent than expected. Thus, both effective and structural connections provide highly plausible routes for non-contiguous seizure spread (Fig. 7, Fig. 9). The high prevalence of both structural and effective connections supports the idea that the seizure spread mechanisms are likely via a direct functional cortico-cortical connection, as opposed to indirect cortico-subcortico-cortical connection.

**Fig. 8:**
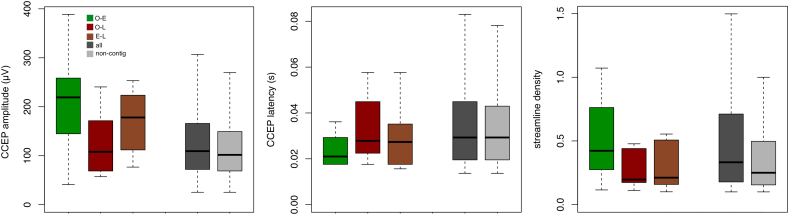
Connection weighting (effective network amplitude and latency, structural network streamline density) of non-contiguous seizure spread connections across all patients. Seizure spread connections (O-E: green, O-L: red and E-L: brown) are compared to the distribution of all connections (dark grey) and all non-contiguous connections (light grey). O-E connections had a higher CCEP amplitude and shorter latency. E-L connections had a higher CCEP amplitude.

There was a trend for higher amplitude of CCEPs from onset to early and early to late sites, but not onset to late seizure spread sites (Fig. 8). As higher amplitude CCEP may reflect a stronger effective connection between those sites, this finding suggests that seizure spread can occur via early propagative sites which act as intermediates site for seizure propagation. The high prevalence of onset to late effective connections suggests that the primary route for seizure spread to late sites is directly from the site of ictal onset. Interestingly, despite high prevalence of all seizure spread routes in the structural networks, there is a trend for lower streamline density for onset to late and early to late connections.

**Fig. 9:**
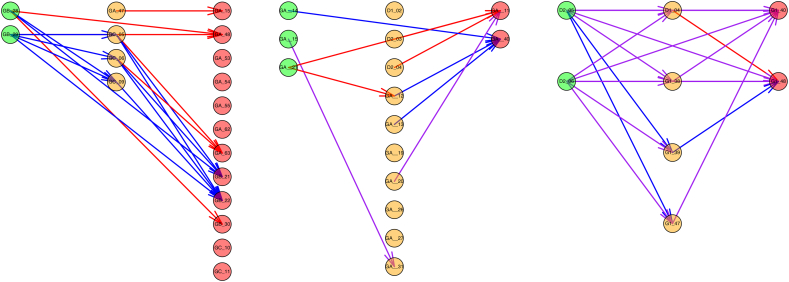
Examples of non-contiguous seizure spread networks in patient 3 (left), 6 (center) and 7 (right). Plausible routes of seizure spread through ictal-onset (green), early propagative (orange) and late propagative (red) contacts were observed when considering evidence of connectivity using either CCEPs (blue), diffusion tractography (red), or both (purple). Patient 3 had no non-contiguous seizure spread connections containing both effective and structural connection, although both modalities individually had connections from both onset to early and early to late propagative contacts, indicating a possible mechanism for non-contiguous seizure spread through intermediate early propagative locations. Patient 6 had connections from onset to early and early to late contacts when considering either or both modalities. Patient 7 had many connections containing both effective and structural connections and many possible routes of seizure spread when considering either or both modalities.

This is the first study to examine mechanisms of non-contiguous seizure spread using CCEPs. Lega et al., (2015) acquired CCEPs in thirty-seven focal epilepsy patients implanted with depth electrodes. The group studied the root-mean-squared (RMS) and gamma band activity in the response period at early and late seizure spread sites. They found an increased RMS and low frequency gamma band activity at early compared to late sites. They hypothesized that low inhibitory input at the early seizure spread sites lead to higher low frequency gamma band activity. These findings of high connectivity at early seizure spread sites concur with our findings of both higher structural and effective connectivity between onset to early sites and supports the notion that seizure spread is via direct effective connection from the ictal-onset zone. A key difference between this study and Lega et al. is that the current study only examined plausible direct seizure spread routes (those from earlier to later propagative sites), whereas Lega et al. examined all CCEPs connecting to early and late sites regardless of their origin. Thus, our findings are more specific in relation to seizure spread whereas the other is more useful for looking at the connectional architecture of seizure spread sites in relation to the rest of the electrode network. In addition, Lega et al. studied epilepsy patients with only depth electrodes implanted, whereas this study includes patients with both grid and depth electrodes, which extends the finding of strongly connected seizure spread sites to cortical as well as sub-cortical sites. A limitation of both studies is the criteria for early and late seizure spread sites. Lega et al. defined early sites as those with seizure activity up to three seconds following seizure onset, and late sites as those with activity greater than three seconds. We used thresholds of less than one second and greater than one second for early and late sites, respectively. The process of identifying seizure contacts is qualitative, and quantitative measures, such as the epileptogenicity index may be less biased and have lower intra-patient variation, which may lead to more accurate assessments of seizure spread (Bartolomei et al., 2008).

## 5 Conclusion

A significant overlap but low correlation was found between structural and effective networks. Low correlation may have been due to the disease-related loss of structure-function correlation or methodological limitations of both modalities and the large-scale nature of the analysis. Altered baseline and effective connectivity was found at ictal-onset contacts, suggesting a hyperexcitable state of cortex at the epileptogenic focus. A high number of outgoing effective connections was found at ictal-onset sites and a high prevalence of structural and effective connections towards sites of non-contiguous seizure spread was observed. Together, these results suggest that the ictal-onset zone is highly excitable and highly outwardly connected, particularly with respect to early and late seizure spread sites. CCEPs may be particularly useful for localising the ictal-onset zone given more widespread disturbances in connection features than in structural networks.

## Funding

This work was undertaken at UCLH/UCL who receive a proportion of funding from the Department of Health NIHR Biomedical Research Centres funding scheme. BD and CS were supported by the NIH-National Institute of Neurological Disorders and Stroke (U01-NS090407-01. The Center for SUDEP Research). SO was supported by the Engineering and Physical Sciences Research Council (EP/L016478/1, NS/A000027/1, EP/M020533/1, EP/N027078/1, NS/A000050/1) and the Wellcome Trust (203145Z/16/Z). RR was supported by Health Innovation Challenge Fund (Welcome Trust and Department of Health; HICF T4-275). Funding sources had no role in study design, collection, analysis or interpretation of data, in the writing of the report; or in the decision to submit the article for publication.

## Appendix A Supplementary data

Methods used to reconstruct effective and structural connectivity of cortical regions underlying intra-cranial electrodes. Effective network reconstruction involved epoching, artefact correction and identifying peaks in the cortico-cortical evoked potential. Structural network reconstruction involved electrode localization, registration to diffusion space and diffusion tractography. Image 1
