# Supplementary material for: Structural and effective connectivity in focal epilepsy
Source: Neuroimage Clin. 2017 Dec 12;17:943–52. doi: 10.1016/j.nicl.2017.12.020 (PMC5842760; doi:10.1016/j.nicl.2017.12.020)
Supplement: Supplementary file 1 — Methods used to reconstruct effective and structural connectivity of cortical regions underlying intra-cranial electrodes. Effective network reconstruction involved epoching, artefact correction and identifying peaks in the cortico-cortical evoked potential. Structural network reconstruction involved electrode localization, registration to diffusion space and diffusion tractography. [file mmc1.pdf]

Supplementary Material

1. Methods

1.1. Electrode Implantation

The decision to implant, the electrode targets, and the duration of implantation was made on clinical grounds, without reference to this study. A total of 644 sub-dural electrodes (grids and strips) and 60 depth electrodes were implanted. Sub-dural electrodes had a diameter of 4 mm, exposed surface area of 2.3 mm and inter-electrode spacing of 10 mm. Some patients had high-density sub-dural grids with 5 mm spacing. Depth electrodes had a diameter of 1.12 mm, length of 2.41 mm and inter-electrode spacing of 10 mm. A small number of depth electrodes were spaced 5 mm apart. Patients were implanted for 5-9 days, so that sufficient monitoring occurred to identify the seizure focus and functionally eloquent cortical areas.

1.2. Reconstructing Effective Networks

1.2.1. Stimulation Parameters

SPES was performed in an electronically shielded room. The NicoletTM Cortical Stimulator with C64-OR amplifiers and Nicolet Cortical Stimulator Control Unit (ISO 13485, ISO 9001; Nicolet Biomedical, Madison, US) was used to deliver a constant-current (AC), bipolar (i.e. between adjacent electrodes), biphasic (500 µs per phase) stimulation pulse of 4 mA intensity at ~0.2 Hz using the Nicolet LTM system (Fig. S1). If clinical signs or afterdischarges were observed, the intensity was reduced to a minimum of 2 mA, in steps of 1 mA. Stimulations were performed using the majority of row-wise adjacent electrode pairs (344 stimulation pairs across all patients). Each stimulation was repeated 10-40 times. Intracranial EEG was recorded using a 128-channel EEG machine (Nicolet Biomedical, Madison, US) with a sampling rate of 512 or 1024 Hz, depending on the number of electrode EEGs acquired (Table S1). An average of two intracranial electrode EEGs with minimal background activity were selected as the reference. These were usually electrodes on sub-dural strips implanted on the inferior surface of the frontal lobe.

Patient	Gender	Age (years)	Epilepsy	Implantation duration (days)	Cortical Dysplasia	Num. Electrodes (analysed/implanted)	Num. Stim. Pairs	Samp. Freq. (Hz)	T1
1	F	49	L IF	5	L IF	62/92	45	1024	QS
2	F	34	L IF	9	L IF	99/104	46	512	C
3	M	26	R FC	8	None	118/128	63	512	C
4	M	39	L F	9	L IF	100/124	55	512	QS
5	F	27	L F	9	None	108/118	52	512	C
6	M	39	L P	7	L P	54/58	28	1024	C
7	M	28	R P	6	None	76/80	51	512	QS

Table S1. Epilepsy patient details. T1 refers to the pre-implantation T1 acquisition site (see section 1.3.1 Image acquisition). Abbreviations: QS = Queen Square, C = Chalfont, L = Left, R = Right, IF = Inferior Frontal, FC = Frontocentral, P = Parietal, F = Frontal

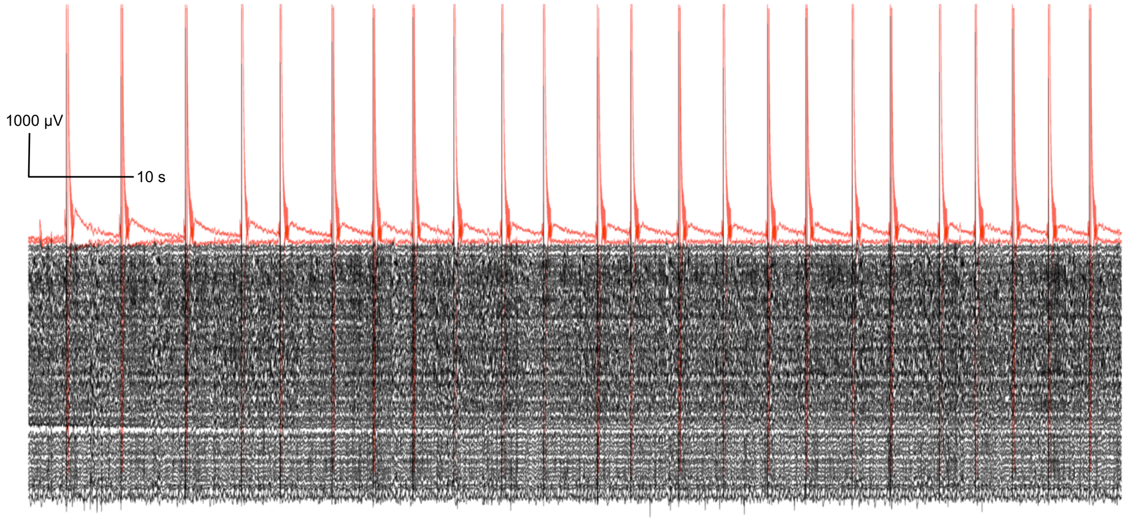

Fig. S1. Raw SPES data for a single stimulation electrode pair. Red and black lines indicate stimulation and recording channels, respectively. Stimulations were delivered approximately once every four seconds. Stimulation delivery times correspond approximately to voltage saturations of the stimulation channels.

1.2.2. Epoching

Epochs were obtained on a stimulation-wise basis. Epoching involved three stages: identifying approximate stimulus delivery time, aligning the stimulus delivery time across stimulation trials, and offsetting the stimulus delivery time of the epoch to the origin (Fig. S2).

SPES data was imported into MATLAB v2011b [1] using EEGLab [2]. MATLAB was used to identify the approximate stimulation onset time (see 1.2.2.1. Identifying Stimulation Ramp-Up Time below). All

subsequent SPES analyses were performed in R v3.2.1 [3].

1.2.2.1. Identifying Stimulation Ramp-Up Time

The anode stimulation channel was thresholded >5000 µV and the first timepoint of each connected component was the initial approximation of the stimulus delivery time. These times correspond to the start of the ramp-up time of the stimulation electrode.

1.2.2.2. Aligning Epochs Across Stimulations

Jitter was observed in the delivery of the stimulus across stimulation trials, with respect to the start of the ramp-up time of the stimulation electrode. This can be seen as a misalignment of the stimulation artefact between stimulation trials, as observed in the nearest recording electrode to the anode. To correct for this, recordings were aligned across stimulations using lagged cross-correlation to the first stimulation in the stimulation train, using a cross-correlation search window of -15:+12 ms. Each stimulation trial was adjusted by the time lag giving the highest correlation to the first stimulation trial.

1.2.2.3. Global Offset of Stimulus Delivery

The stimulus delivery did not immediately follow the start of the voltage ramp-up of the stimulation channel (Fig. S2). Therefore, a global offset was applied to all recording channels for all stimulations. The global offset was calculated as the time until maximum amplitude of the mean signal following ramp-up start time within a window of -15:+12 ms following ramp-up at the nearest recording electrode. An epoch was generated for each stimulation and consisted of a three-dimensional array containing the amplitudes at timepoint indices for each recording electrode.

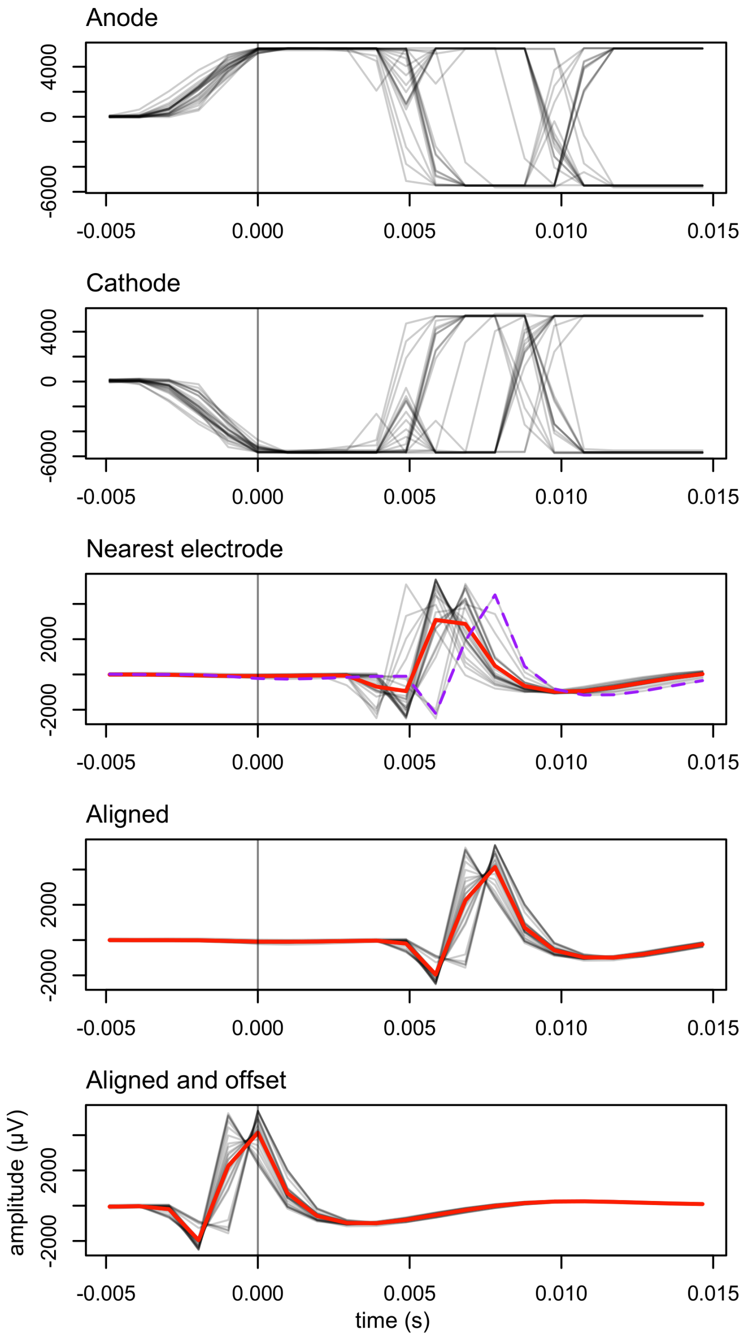

Fig. S2. Summary of SPES epoching. For each stimulation a current is generated between the anode and cathode electrodes, which become positively and negatively charged, respectively. Saturation of the anode channel was used as an initial estimate of the stimulus delivery time (time point 0). The artefact was prominent on the nearby recording electrodes and was a better estimate of the stimulus delivery time. All stimulations were aligned to the first stimulation (purple line, Nearest electrode) using cross-correlation of the amplitude in the electrode nearest to the anode. The aligned data was then offset to time point 0 using the maximum amplitude of the mean (red lines) of the electrode nearest the anode channel. This example stimulation is between two depth electrodes in patient 1.

1.2.3. Artefact Correction

The signal amplitude in recording electrodes immediately following stimulation depended on the charge polarity of the nearest stimulation electrode. Therefore, an ICA-based artefact reduction technique was implemented, whereby the data was re-projected following removal of the independent components (ICs) of the signal representing the stimulation artefact. This is similar to previous artefact reduction techniques applied to scalp EEG [4] and transcranial magnetic stimulation (TMS).

ICA was performed for each stimulation trial separately using only the amplitudes of the recording electrodes. The fast-ICA algorithm was used to decompose the observed signals into five ICs within a time period of -40:+40 ms using the fastICA function in R [5]. The artefactual ICs were selected automatically, as those with absolute maximum above two standard deviations of the median of the absolute maximums across all ICs. The ICs representing artefact were removed from the source signal matrix and the signals were then reconstructed by projecting the remaining ICs in the signal matrix using the ICA mixing matrix.

Two artefactual ICs were most commonly designated as artefact. Increasing or decreasing the number of initial ICs had little effect on the number of ICs representing stimulation artefact.

1.2.4. Visually Excluding Other Artefacts

Following artefact correction, the mean signal of every recording electrode for every stimulation was visually examined to identify further artefacts. Epochs containing artefacts were excluded from all future analyses. In total 673/30198 epochs were excluded due to artefacts. Artefacts were either (i) non-physiological ramp-shaped response following stimulation (ii) large voltage surges (iii) visible 50 Hz power line noise in the average response, due to a combination of both 50 Hz power line noise contamination and a low number of stimulation trials (iv) slow exponential return to baseline following stimulation, indicating bad contacts.

1.2.5. Cortico-Cortical Evoked Potential Peaks

An in-house peak finding algorithm was implemented on the mean signal of each recording electrode to identify amplitudes and latency of evoked potentials. Firstly, amplitudes were low-pass filtered using a Butterworth filter with an order of 5 ms and a frequency of 110 Hz. The frequency of the filter was set to allow fast evoked potentials to pass whilst removing high frequency noise which may lead to false positive peak detection. Secondly, candidate peaks were identified by selecting those timepoints surrounded by positive and negative gradient. Next, candidate peaks were grouped by the connected components of the signal with low gradient (mean gradient <2 uV/ms within a 5 ms neighbourhood). The candidate peaks with maximum absolute amplitude in each connected component were selected. Finally, those selected candidate peaks with amplitude below twice the baseline standard deviation were excluded. In this calculation the standard deviation of the baseline was calculated using all timepoints across all stimulation trials in the time period of -500:-15 ms, whereas the amplitude searched for CCEPs was that of the mean evoked potential peak in the time period of +12:+250 ms. Positive amplitude peaks with a prior negative gradient and negative amplitude peaks with a prior positive gradient were removed.

1.2.6 Effective Networks

An evoked waveform represents a connection between the stimulation electrode(s) and the recording electrode. The evoked potential may arise from a connection between the stimulation-electrode pair to the recording electrode, or from either stimulation electrode to the recording electrode. In consideration of these different methods to interpret evoked waveforms, peak features of interest were converted from the original to network format. The original representation is the native format of the CCEP data after peak-finding: each observed peak represents a single data point corresponding to a connection between the stimulating electrode pair and the recording electrode. In the network representation, we reconstructed the electrode-electrode connectivity using the original CCEP data. In the network representation, each peak represents two connections in the electrode-electrode network; one from each stimulation electrode to the recording electrode. Note that in cases where responses had multiple peaks, only

the first peak was used. Peak features of stimulation electrode-recording electrode pairs involved in multiple stimulations were averaged. The network representation therefore involves duplication and some averaging of peak amplitudes. The network representation resulted in a directed electrode-electrode network of peak amplitudes and permitted large-scale network analysis and direct connection-wise comparison of connections between effective and structural networks in the native structural network format.

1.3. Reconstructing Structural Networks

1.3.1. Image Acquisition

Pre-implantation T1 and diffusion weighted images and post-implantation T1 and CT were acquired for routine clinical assessment. Pre-implantation diffusion weighted sequences were acquired using a single-shot spin-echo planar imaging (EPI) sequence, cardiac gated with TE = 73 ms. Sets of 60 contiguous 2.4 mm thick axial slices were obtained, covering the whole brain, with diffusion-sensitizing gradients applied in each of 52 non-collinear directions (maximum b-value of 1200 mm^2 s^{-1} [δ = 21 ms, Δ = 29 ms, using a gradient strength of 40 mT m^{-1}]) along with six non-diffusion-weighted (b = 0) scans. The gradient directions were calculated and ordered as described elsewhere [6]. The parallel imaging factor (SENSE) was 2. The field of view was 24 cm, and the acquisition matrix size was 96 x 96, zero filled to 128 x 128 during reconstruction so that the reconstructed voxel size was 1.875 x 1.875 x 2.4 mm. The DTI acquisition time was ~25 min, depending on patient heart rate.

One of two pre-implantation T1-weighted sequences (either 'Queen Square' or 'Chalfont',) were acquired for each patient, depending on the MRI centre attended at the time of evaluation (see Table S1). 3/7 patients (1,4 and 7) had Gradient Recalled Inversion Recovery sequences at 1.5T on a Siemens Avanto scanner at the Neuroradiology Department, NHNN, Queen Square. These images were 0.488 x 0.488 x 1.500 mm resolution, acquired with 144 axial orientated slices of 249.856 mm^2 using an acquisition matrix of 512 by 512. The TR/ TE/ flip-angle was 2020 ms/1.71 ms/15°. 4/7 patients (2,3,5 and 6) had Gradient Recalled sequence at 3T on a GE Medical Systems MR750 scanner at the Chalfont MRI Unit, Epilepsy Society, Chalfont St. Peter.

These images were 0.938 x 0.938 x 1.100 mm resolution, acquired with either (i) 170 coronal orientated slices (3/4 patients) of 240.128 mm^2 using an acquisition matrix of 256 by 256, or (ii) 160 axial orientated slices (1/4 patients), each of 240.128 x 242.004 mm, using an acquisition matrix of 256 by 258. The TR/TE/flip-angle was 7.96 ms/3.008 ms/20°.

Post-implantation T1-weighted images were acquired using a Gradient Recalled Inversion Recovery sequence at 1.5T on a Siemens Avanto scanner at the Neuroradiology Department, NHNN. These images were of 1 x 1 x 1 mm resolution, acquired with 176 coronal-orientated slices of 192 by 192 voxels. The TR/TE/flip-angle was 1930 ms/ 3.37 ms/ 15◦.

Post-implantation volumetric brain CT images of 0.43 x 0.43 x 1 mm resolution were acquired in axial orientation.

1.3.2. Electrode Localisation in Diffusion Space

Electrode voxel co-ordinates were calculated from the CT by plotting and manually labelling supra-threshold voxel clusters with reference to the patient implantation notes. Electrodes invisible on CT or absent intracranial recordings were not analysed (87/704 implanted).

Estimating structural connectivity between electrodes requires transformation of electrodes from CT to diffusion space and estimation of the underlying brain region. To account for substantial brain shift following electrode implantation, non-rigid co-registrations were applied between pre-implantation and post-implantation spaces. Firstly, a rigid registration was optimised between the post-implantation CT and post-implantation T1 image. A non-rigid registration was then optimised between the post-implantation T1 and pre-implantation T1 to correct for brain shift, and between pre-implantation T1 and diffusion (first b=0 image) images. The transformation field required to transform electrode co-ordinates from CT to diffusion space was composed using the following transformation fields: (i) post-implantation CT to post- implantation T1 (ii) post-implantation T1 to pre-implantation T1 and (iii) pre-implantation T1 to pre-implantation diffusion. All registrations, compositions and transformations were implemented in NiftyReg (v1.3.9) using the default settings [7]. NiftyReg uses normalised mutual information to calculate image similarity and a

bending energy regularisation with cubic B-spline parameterisation for the non-linear warping.

1.3.3. Diffusion Tractography

Pre-implantation T1 images were parcellated into cortical and sub-cortical grey matter, white matter and ventricles using the LoAd tissue segmentation algorithm implemented in NiftySeg [8]. Parcellations were transformed to diffusion space using the aforementioned rigid transformation obtained between the pre-implantation T1 and diffusion image (see section 1.3.2. Electrode Localisation in Diffusion Space). A nearest neighbour resampling scheme was used to preserve the categorical nature of parcellation labels. The tissue parcellation was used to define seed, propagation, and termination masks for fiber tractography. These masks define the starting location, permitted tractography region, and termination regions for local fiber tractography. Firstly, cortical grey matter was assigned to the nearest electrode voxel within a maximum of 10 mm and depth electrodes voxels were dilated by 5 mm to create the electrode parcels. Any parcels that overlapped were assigned to the nearest electrode. The binary version of these cortical parcels was the termination mask. The intersection of the dilated termination mask and the white matter parcellation defined the seed mask. The propagation mask was the union of white matter, sub-cortical grey matter and ventricle regions from the parcellation. The termination mask was mutually exclusive from both the propagation and seed masks.

Next, fiber paths in the brain were reconstructed by seeding 100 probabilistic fibers from each seed voxel using MRTrix. Fibers were propagated using the default settings in MRTrix [9]. The sampling interval was 0.2 mm, maximum curvature threshold was 60° and minimum fiber orientation dispersion (FOD) amplitude threshold for tracking through a voxel was 0.1. Fiber propagation was stopped when exiting the propagation mask or entering the termination mask.

1.3.4. Structural Networks

Inter-electrode structural networks were obtained by representing electrode parcels as network nodes and the fiber tractography connections between them as edges. Fibers connected node pairs if

their end-point coordinates terminated within two distinct electrode cortical parcels. The connection weight between two cortical nodes was defined as the density of connecting fibers (as in [10]), calculated as the sum of connecting fibers divided by the mean volume of the seed (boundary) voxels adjacent to the two parcels (boundary voxels were assigned to the nearest parcel by Euclidean distance). This resulted in a N-by-N connectivity matrix of fiber densities between all N electrode parcels. We considered connections with streamline density above 0.1 in this report, for lack of a standardised thresholding method.

References

[1] MATLAB. version 7.10.0 (R2010a). The MathWorks Inc., Natick, Massachusetts, 2010.

[2] A. Delorme and S. Makeig. Eeglab: an open source toolbox for analysis of single-trial eeg dynamics including independent component analysis. Journal of neuroscience methods, 134(1):9–21, 2004.

[3] R Development Core Team. R: A Language and Environment for Statistical Computing. R Foundation for Statistical Computing, Vienna, Austria, 2008. URL http://www.R-project.org. ISBN 3-900051-07-0.

[4] A. Delorme, T. Sejnowski, and S. Makeig. Enhanced detection of artifacts in eeg data using higher-order statistics and independent component analysis. Neuroimage, 34(4):1443–1449, 2007.

[5] A. Hyvärinen and E. Oja. Independent component analysis: algorithms and applications. Neural networks, 13(4):411–430, 2000.

[6] P. A. Cook, M. Symms, P. A. Boulby, and D. C. Alexander. Optimal acquisition orders of diffusion-weighted mri measurements. Journal of magnetic resonance imaging, 25(5):1051–1058, 2007.

[7] M. Modat, G. R. Ridgway, Z. A. Taylor, M. Lehmann, J. Barnes, D. J. Hawkes, N. C. Fox, and S. Ourselin. Fast free-form deformation using graphics processing units. Comput. Methods Prog. Biomed., 98(3):278–284, 2010.

[8] M. J. Cardoso, M. J. Clarkson, G. R. Ridgway, M. Modat, N. C. Fox, and S. Ourselin. Load: A locally adaptive cortical segmentation algorithm. NeuroImage, 56(3):1386–1397, 2011.

[9] J. Tournier, F. Calamante, and A. Connelly. Mrtrix: Diffusion tractography in crossing fiber regions. Int. J. Imaging Syst. Technol., 22(1): 53–66, 2012. 12

[10] H. Cheng, Y. Wang, J. Sheng, O. Sporns, W. G. Kronenberger, V. P. Mathews, T. A. Hummer, and A. J. Saykin. Optimization of seed density in DTI tractography for structural networks. J. Neurosci. Methods, 203 (1):264–272, 2012.
